# Evaluation of programmed cell death ligand-1 expression in primary central nervous system lymphoma using whole-tumor histogram analysis of multiparametric MRI: implications for immunotherapy selection

**DOI:** 10.3389/fimmu.2025.1676273

**Published:** 2025-12-11

**Authors:** Xiaofang Zhou, Xiaoli Su, Lan Yu, Feng Wang, Shujie Yu, Feifei Yu, Xiaoye Lin, Yang Song, Dairong Cao, Xingfu Wang, Zhen Xing

**Affiliations:** 1 Department of Radiology, The First Affiliated Hospital, Fujian Medical University, Fuzhou, China; 2 Department of Radiology, National Regional Medical Center, Fujian Medical University, Fuzhou, Fujian, China; 3 Department of Pathology, The First Affiliated Hospital, Fujian Medical University, Fuzhou, China; 4 Department of Pathology, National Regional Medical Center, Fujian Medical University, Fuzhou, Fujian, China; 5 MR Research Collaboration Team, Siemens Healthineers Ltd., Shanghai, China; 6 Department of Radiology, Fujian Key Laboratory of Precision Medicine for Cancer, The First Affiliated Hospital, Fujian Medical University, Fuzhou, China; 7 Key Laboratory of Radiation Biology of Fujian Higher Education Institutions, The First Affiliated Hospital, Fujian Medical University, Fuzhou, China; 8 Department of Pathology, Jianning General Hospital, Sanming, Fujian, China

**Keywords:** primary central nervous system lymphoma, programmed cell death ligand-1, magnetic resonance imaging, whole-tumor histogram analysis, immunotherapy

## Abstract

**Objective:**

To assess the diagnostic performance of whole-tumor histogram analysis of multiparametric MRI in predicting programmed cell death ligand-1 (PD-L1) expression in primary central nervous system lymphoma (PCNSL).

**Methods:**

A total of 130 patients with PCNSL (61 males, aged 21–80 years) were included in the study. Histogram features derived from T2-weighted imaging (T2WI), T1-weighted imaging (T1WI), fluid-attenuated inversion recovery (FLAIR), contrast-enhanced T1-weighted imaging (T1WI+C), and apparent diffusion coefficient (ADC) were compared between the low and high PD-L1 expression groups using the Mann-Whitney U test. Receiver operating characteristic (ROC) curves and logistic regression analysis were applied to assess the diagnostic performance of both individual and combined models in predicting PD-L1 expression levels in PCNSL.

**Results:**

Eighteen histogram features extracted from multiparametric MRI exhibited significant differences between high and low PD-L1 expression in PCNSL (all *P* < 0.05). The predictive performance of single-sequence models was relatively modest, with areas under the curve (AUC) ranging from 0.637 to 0.705, and no significant differences were observed between these models (all *P* > 0.05). The combined model demonstrated the highest diagnostic performance (AUC = 0.809), significantly outperforming the single-sequence models (all *P* < 0.05).

**Conclusions:**

Whole-tumor histogram analysis of multiparametric MRI shows potential as a non-invasive method for evaluating PD-L1 expression in PCNSL, which may assist in the identification of immunotherapy-eligible patients.

## Highlights

- Early non-invasive assessment of PD-L1 expression is critical for personalized immunotherapy.
- Multiparametric MRI-based whole-tumor histogram analysis effectively assesses PD-L1 expression in PCNSL.
- The combined model offers the highest diagnostic performance, aiding clinicians in immunotherapy decision-making for patients with PCNSL.

## Introduction

Primary central nervous system lymphoma (PCNSL) is a rare and highly aggressive type of non-Hodgkin lymphoma, characterized by challenging treatment, high relapse rates, and poor prognosis, accounting for nearly 5% of all recently identified intracranial neoplasms (1). Although first-line chemotherapy based on high-dose methotrexate can improve patient outcomes, 30-40% of patients experience relapse (2). In recent years, with advances in molecular biology, novel immune checkpoint inhibitors targeting the programmed cell death-1 and its ligand (PD-L1) pathway have emerged as promising therapeutic options for PCNSL (3). However, these therapies are associated with severe toxicities, and not all patients benefit from them. Multiple studies have demonstrated that PD-L1 expression in tumor cells serves as an independent predictive biomarker for both prognosis and therapeutic efficacy, making it the most widely adopted biomarker for optimizing patient selection (4–7). Currently, PD-L1 detection primarily relies on invasive biopsy, which is not only traumatic but also unable to fully represent the overall characteristics of the tumor and challenging to monitor dynamically. Therefore, there is an urgent need for developing non-invasive, easily accessible pre-treatment imaging biomarkers.

Multiparametric magnetic resonance imaging (MRI), including T2-weighted imaging (T2WI), T1-weighted imaging (T1WI) with or without contrast, and diffusion-weighted imaging (DWI), provides non-invasive and critical insights into tumor morphology and molecular characteristics. However, our prior research has demonstrated that, while traditional imaging techniques offer some information regarding tumor size and morphological changes, their capacity to predict PD-L1 expression in PCNSL remains limited (8). Advanced imaging analysis techniques, such as histogram analysis and radiomics, enable the extraction of high-dimensional features from imaging data, which can capture tumor biological characteristics not readily detectable through conventional imaging methods. These approaches have shown promise in predicting PD-L1 expression and responses to immunotherapy in other cancers (9–12). However, due to the intricate nature of radiomics, its implementation in routine clinical practice remains challenging. In comparison, whole-tumor histogram analysis offers a simpler approach and demonstrates greater feasibility for clinical application. To date, and to the best of our knowledge, no studies have investigated the potential of multiparametric MRI-based histogram analysis for evaluating PD-L1 expression in PCNSL.

Therefore, this study aims to explore the predictive efficacy of whole-tumor histogram analysis based on multiparametric MRI in evaluating PD-L1 expression in patients with PCNSL, with the goal of providing valuable insights for optimizing treatment strategies.

## Materials and methods

### Patients

**Figure 1:**
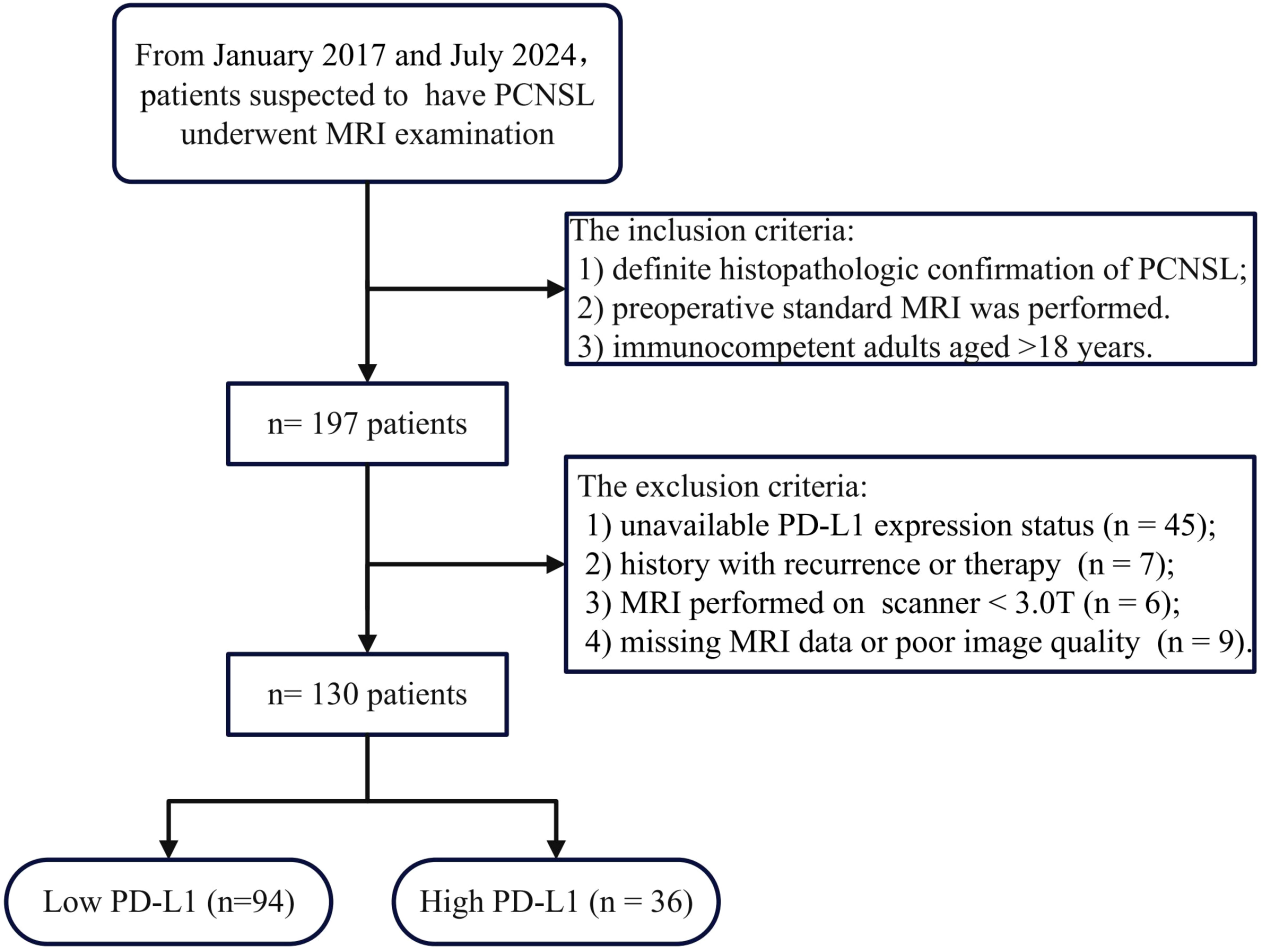
Patient flowchart for this study.

The ethics committee of our hospital approved this retrospective study and patient informed consent was waived. A total of 197 patients with histologically confirmed PCNSL were consecutively enrolled between January 2017 and July 2024, in accordance with the inclusion criteria listed below: 1) definitive diagnosis of PCNSL was made through histopathological evaluation of tissue acquired by stereotactic or surgical methods, as per the 2021 WHO criteria.; 2) standard preoperative MRI protocols were conducted, comprising T2WI, T1WI, T1WI with contrast (T1WI+C), Fluid-attenuated inversion recovery (FLAIR), and DWI; 3) immunocompetent adults aged >18 years. The exclusion criteria were as follows:1) undetermined PD-L1 expression levels (n=45); 2) history of any prior treatment or recurrence before undergoing MRI(n=7). 3) MRI conducted on a scanner with a magnetic field strength of below3.0T (n = 6); 4) missing MRI data or poor image quality (n = 9). Ultimately, 130 patients were included in this study. The process of patient enrollment is outlined in **Figure 1**.

### MRI protocols

All MRI acquisitions were conducted on 3.0 T scanners (Verio/Skyra/Prisma; Siemens Healthcare, Erlangen, Germany). The morphological MRI (mMRI) protocol consisted of axial T2WI (TR/TE 4000/125 ms), T1WI (TR/TE 250/2.48 ms), FLAIR (TR/TE 9000/94 ms), and T1WI+C (TR/TE 250/2.48 ms). Uniform acquisition parameters were maintained across sequences: 220 × 220 mm field-of-view (FOV), 5 mm slice thickness, 256 × 256 acquisition matrix, and 1 mm interslice gap, in accordance with our prior investigations (8). DWI acquisitions utilized an axial echo-planar sequence applied in all three orthogonal directions. The technical parameters comprised: b-values 0/1000 s/mm², TR/TE 8200/102 ms, acquisition matrix 128×128, FOV 220×220 mm², 2 excitations, 5 mm slice thickness, and 1 mm interslice gap. Automated reconstruction of apparent diffusion coefficient (ADC) map was conducted using dedicated MRI postprocessing platforms through standardized computational protocols. The MRI protocols are summarized in **Table 1**.

**Table 1:** MRI protocols.

Sequences	T2WI	T1WI	FLAIR	T1WI+C	DWI
TR/TE (ms)	4000/125	250/2.48	9000/94	250/2.48	8200/102
FOV (mm)	220 × 220	220 × 220	220 × 220	220 × 220	220 × 220
Slice thickness (mm)	5	5	5	5	5
Matrix size	256 × 256	256 × 256	256 × 256	256 × 256	128 ×128
Slice gap (mm)	1	1	1	1	1
b Values (s/mm2)	–	–	–	–	0/1000

TR, repetition time; TE, echo time; FOV, field of view; NEX, number of excitations; T2WI, T2-weighted imaging; T1WI, T1-weighted imaging; DWI, Diffusion-weighted imaging; T1WI+C, Contrast-enhanced T1-weighted imaging; FLAIR, Fluid-attenuated inversion recovery

### Image processing and analysis

Before preprocessing, the DICOM images were initially converted to NIfTI format. The T1WI, T1WI+C, FLAIR, and ADC sequences were subsequently co-registered to the T2WI using SPM8 software. This registration employed a normalized mutual information cost function and cubic B-spline interpolation (http://www.fil.ion.ucl.ac.uk/spm/).

**Figure 2:**
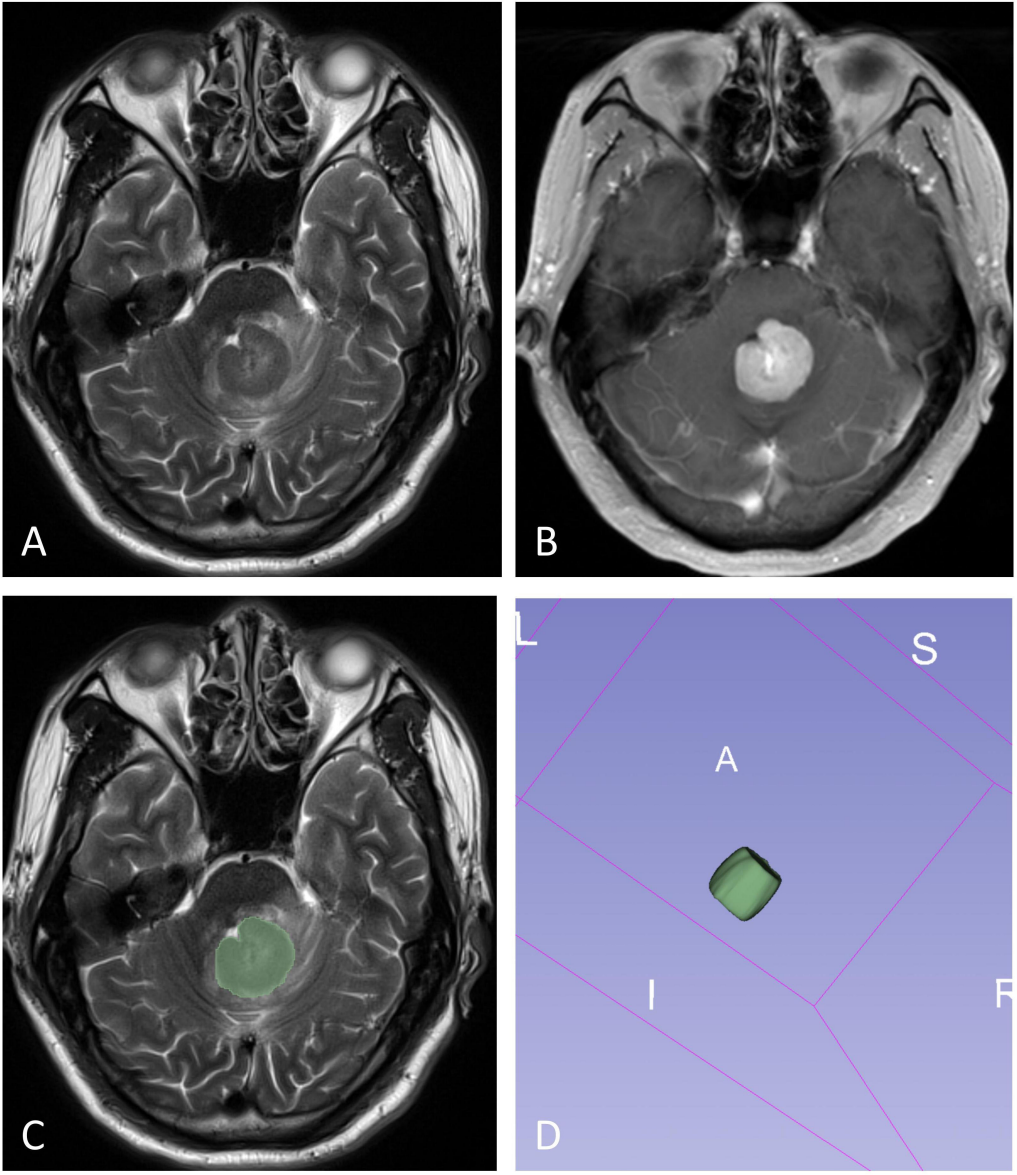
The illustration for VOI selection in PCNSLs. ROIs (green line) were drawn over the solid component of the tumors on each section **(C)**, with reference to T2WI **(A)** and contrast-enhanced T1WI **(B)**. The final VOI was then generated **(D)**.

The volume of interest (VOI) was manually delineated by two neuroradiologists (X.Z. and L.Y., with 6 and 2 years of neuroradiology experience, respectively) blinded to PD-L1 expression through consensus. In cases of disagreement, a senior radiologist (D.C, with 32 years of neuroradiology experience) made the final determination. The VOI was carefully outlined along the solid tumor margin on the T2WI and T1WI+C images, with the exclusion of cystic components, necrotic areas, hemorrhages and blood vessels (**Figure 2**). These VOIs, initially defined on the T2WI and T1WI+C images, were then transferred to the T1WI, FLAIR, and ADC images. VOI delineation was performed using the open-source software tool 3D Slicer (version 4.13.0; https://www.slicer.org/).

We analyzed each sequence for all cases based on the Image Biomarker Standardization Initiative. The process and parameters are outlined below. Considering that the imaging sequence was acquired in 2D, there was a notable difference between the in-plane and through-plane resolutions. Histogram-based features were extracted from the 3D volume analysis. All cases were first resampled to an in-plane resolution of (0.69, 0.69), and the VOIs were re-segmented using the 3-sigma method. As the sequence was non-quantitative, the data for each case were normalized individually using Z-scores. Histogram features were then directly extracted from the volume data. Histogram features were extracted using FeAture Explorer, an open-source analysis tool (FAE, version 0.5.5; https://github.com/salan668/FAE). The histogram features obtained through this software were as follows: 10th percentile, 90th percentile, Energy, Entropy, Interquartile Range, Kurtosis, Maximum, Mean Absolute Deviation, Mean, Median, Minimum, Range, Robust Mean Absolute Deviation, Root Mean Squared, Skewness, Uniformity, Variance.

### Histopathological and immunohistochemical evaluation

The histopathological diagnoses for all cases were independently assessed by a pathologist with 23 years of extensive experience in neuropathological diagnosis. PD-L1 expression was assessed by immunohistochemistry on formalin-fixed, paraffin-embedded specimens using the PD-L1 IHC 28–8 pharmDx kit (monoclonal mouse anti-human antibody, clone 28-8; Abcam PLC, UK). According to previous studies (4), PD-L1 expression levels were stratified based on the proportion of PD-L1-positive tumor cells: high expression was defined as >30%, and low expression as ≤30% of total tumor cells.

### Statistical analysis

All data were presented as medians (interquartile range), means (standard deviation), or number of cases, as appropriate. Continuous variables in the clinicopathological features were compared using t-tests, while categorical variables were analyzed with chi-square tests. Histogram data obtained from multiparametric MRI sequences were subjected to the Mann-Whitney U test to compare differences between low and high PD-L1 expression. Statistically significant variables associated with PD-L1 expression were selected for binary logistic regression analysis to construct both individual and combined models. A backward stepwise selection procedure was applied with p-values for entry and removal set at p<0.05 and p>0.1, respectively. The model's overall significance was assessed using the Omnibus tests of model coefficients. The Hosmer–Lemeshow test was employed to evaluate goodness-of-fit. The diagnostic performance of the different models in distinguishing low and high PD-L1 status was evaluated using receiver operating characteristic (ROC) curve analysis. Comparisons of the area under the curve (AUC) between models were conducted using the DeLong test. A p-value less than 0.05 was considered statistically significant. All statistical analyses were performed using IBM SPSS Statistics (version 25.0) and MedCalc (version 15.6).

## Results

A total of 130 patients (61 males, 69 females; mean age 59.56 ± 11.01 years; age range 21–80 years) were included in this study. According to immunohistochemical findings, 94 patients with PCNSL (72.3%) were classified as low PD-L1 expression, while 36 patients (27.7%) were classified as high PD-L1 expression. No statistically significant differences were observed in age, gender, Hans classification, or Ki-67 index between PCNSL patients with low versus high PD-L1 expression. The clinicopathological characteristics of the study cohort are summarized in **Table 2**.

**Table 2:** Patients baseline clinicopathological characteristics.

Parameters	Low PD-L1	High PD-L1	P value
No. of patients	94	36	
Mean age and SD (y)	60 ± 11	57 ± 10	0.240
Gender			0.107
Male	40	21	
Female	54	15	
Hans type			0.398
GCB	8	7	
Non-GCB	41	22	
Ki-67 index (mean ± SD)	83 ± 11	83 ± 13	0.901

GCB, Germinal Center B-cell-like; Non-GCB, Non-Germinal Center B-cell-like.

**Figure 3:**
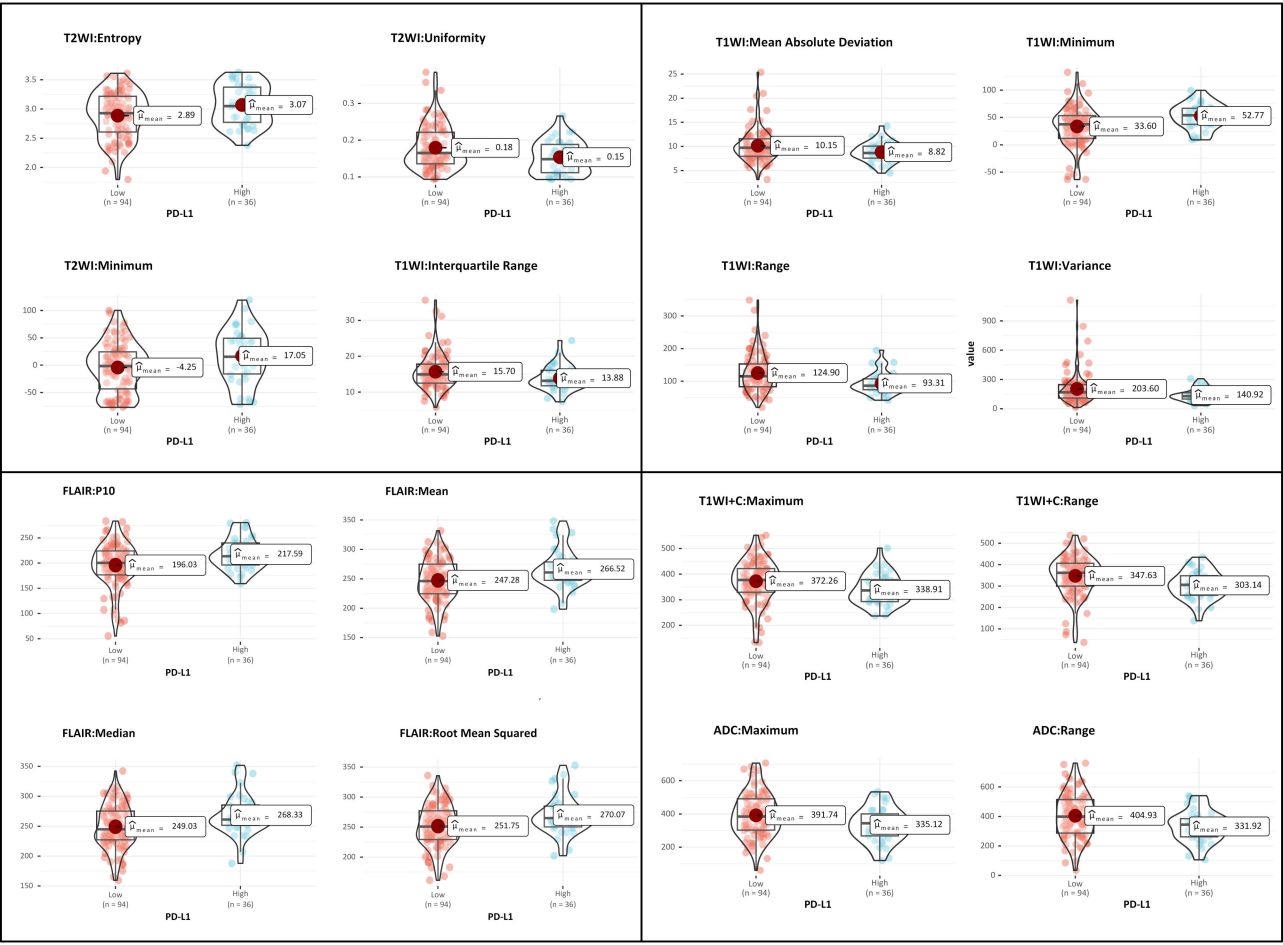
Violin plots show histogram features of multiparametric MRI between different PD-L1 status of PCNSL.

The comparison of all histogram features derived from multiparametric MRI between the high and low PD-L1 expression groups is summarized in **Supplementary Table S1**. Histogram features exhibiting statistically significant differences between the two groups are presented in **Table 3** and **Figure 3**.

**Table 3:** Comparisons of histogram features of multiparametric MRI between PCNSLs with low and high PD-L1 expression.

Parameters	Low PD-L1	High PD-L1	P value
T2WI model			
Entropy	2.89 ± 0.40	3.07 ± 0.34	0.026
Minimum	-4.25 ± 45.26	17.05 ± 49.53	0.031
Uniformity	0.18 ± 0.06	0.15 ± 0.05	0.034
T1WI model			
Interquartile Range	15.70 ± 4.98	13.88 ± 3.45	0.040
Kurtosis	5.89 ± 5.09	4.63 ± 2.90	0.046
Mean Absolute Deviation	10.15 ± 3.42	8.82 ± 2.01	0.033
Minimum	33.60 ± 35.47	52.77 ± 22.45	0.001
Range	124.90 ± 58.16	93.31 ± 33.13	0.002
Variance	203.60 ± 162.20	140.92 ± 65.18	0.018
FLAIR model			
P10	196.03 ± 44.29	217.59 ± 30.50	0.016
P90	299.53 ± 39.33	316.74 ± 36.50	0.045
Mean	247.28 ± 35.80	266.52 ± 32.58	0.011
Median	249.03 ± 36.25	268.33 ± 34.30	0.008
Root Mean Squared	251.75 ± 35.03	270.07 ± 32.55	0.013
T1WI+C model			
Maximum	372.26 ± 82.95	338.90 ± 60.09	0.007
Range	347.63 ± 93.17	303.14 ± 65.97	0.002
ADC model			
Maximum	391.74 ± 131.25	335.12 ± 103.38	0.027
Range	404.93 ± 151.31	331.92 ± 105.13	0.011

P10, 10th percentile; P90, 90th percentile

**Figure 4:**
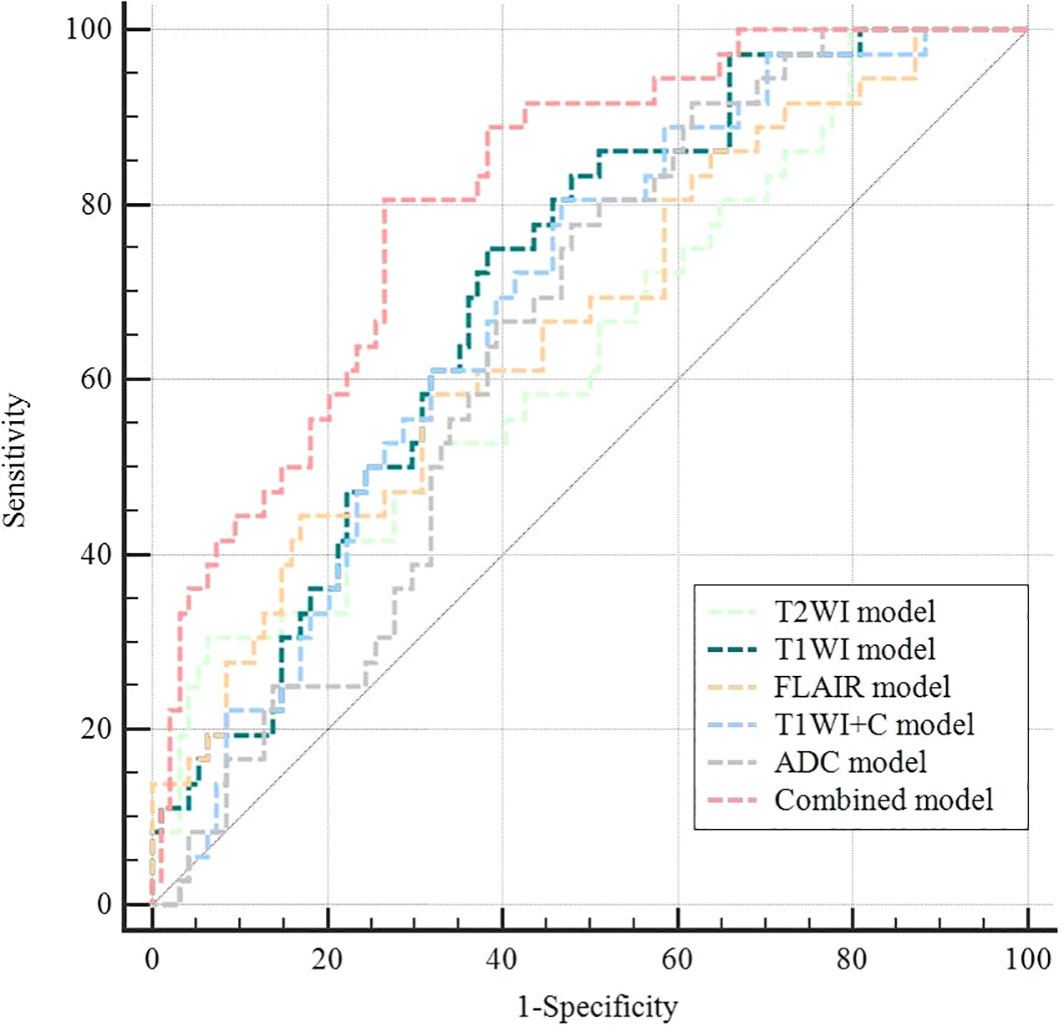
ROC curves of different models in predicting PD-L1 status of PCNSL.

**Figure 5:**
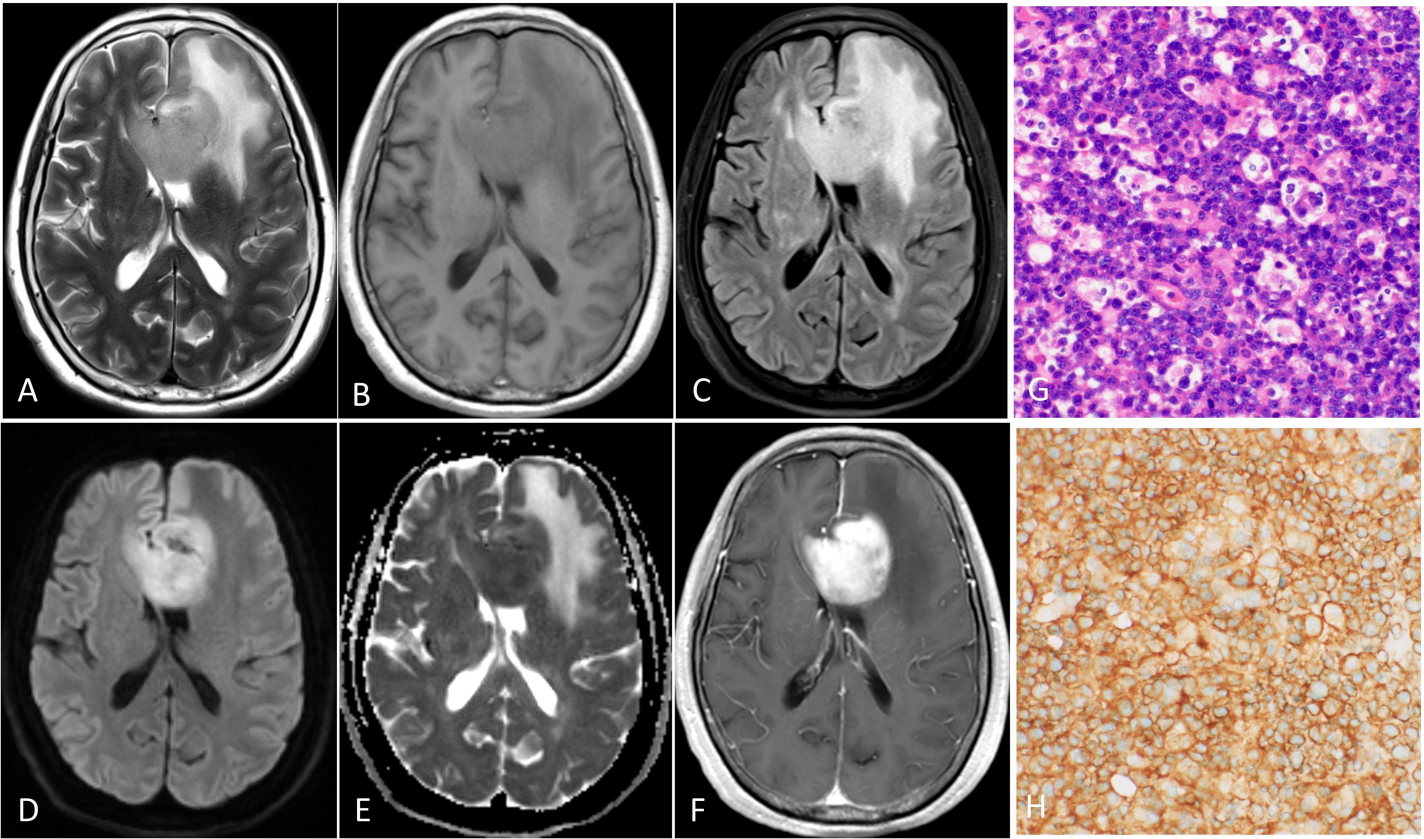
PCNSL with high PD-L1 expression in the corpus callosum. The tumor demonstrates homogeneous obviously hyperintensity on T2WI **(A)**, FLAIR **(C)** and DWI **(D)**, slightly hypointensity on the T1WI **(B)** and obviously hypointensity on ADC map **(E)**, and marked enhancement on the contrast-enhanced T1WI **(F)**. **(G)** Hematoxylin-eosin staining confirms the mass as a diffuse large B cell lymphoma. **(H)** PD-L1 immunohistochemical test shows that approximately 95% of positive tumor cells for staining. (magnification, × 40).

**Figure 6:**
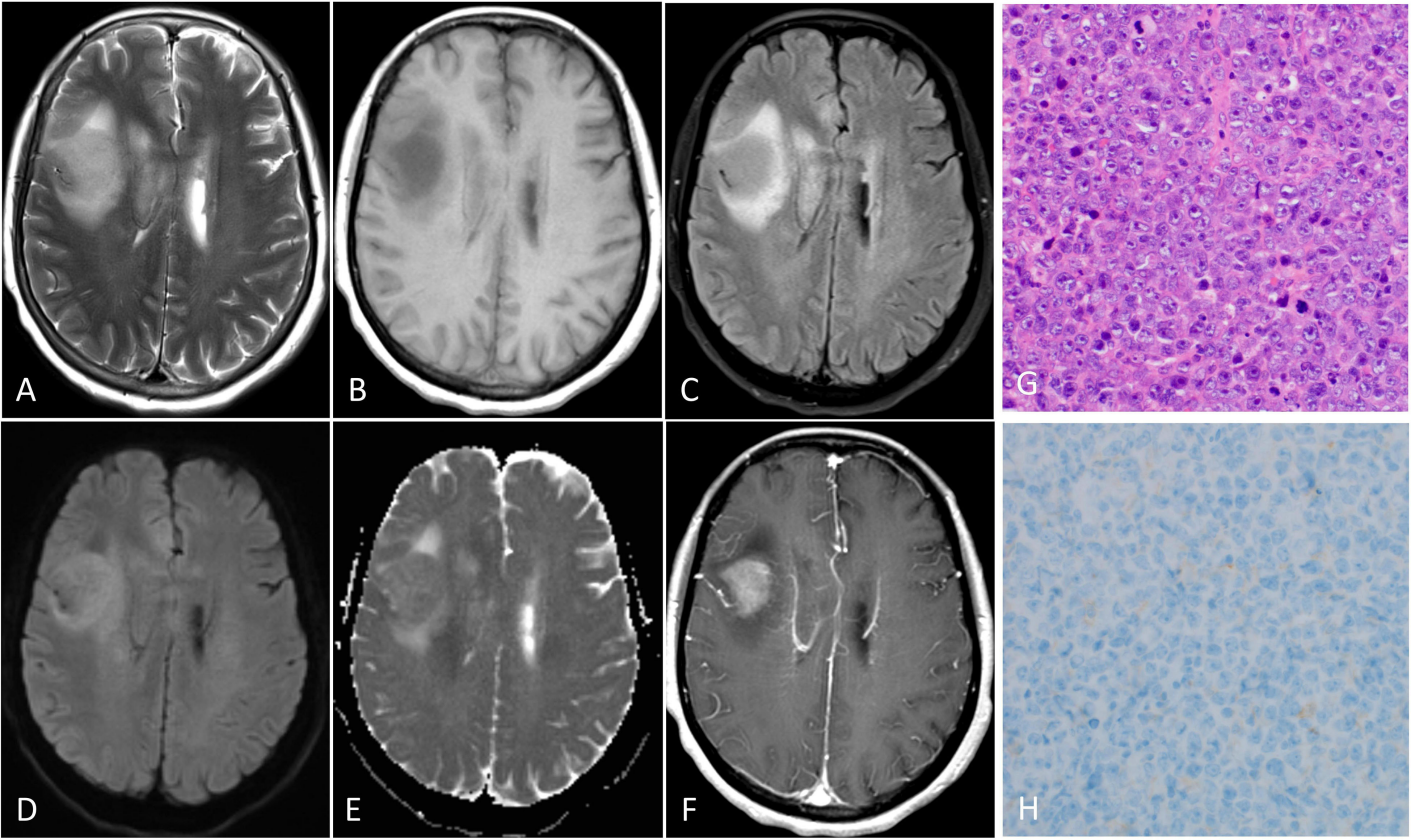
PCNSL with low PD-L1 expression in the right frontal lobe. The tumor demonstrates homogeneous moderately hyperintensity on T2WI **(A)**, FLAIR **(C)** and DWI **(D)**, obviously hypointensity on T1WI **(B)** and slightly hypointensity on ADC map **(E)**, and moderately enhancement on the contrast-enhanced T1WI **(F)**. **(G)** Hematoxylin-eosin staining confirms the mass as a diffuse large B cell lymphoma. **(H)** PD-L1 immunohistochemical test shows that approximately 2% of positive tumor cells for staining. (magnification, × 40).

All models were statistically significant (Omnibus test p < 0.05) and demonstrated good fit (Hosmer-Lemeshow test p > 0.05). The logistic regression analysis revealed that T2WI_Entropy, T1WI_Interquartile Range, T1WI_Mean Absolute Deviation, T1WI_Minimum, T1WI+C_Range, ADC_Range, and FLAIR_Median were independent predictors of PD-L1 expression status. The diagnostic performance of individual models, including T2WI, T1WI, T1WI+C, FLAIR, and ADC, in discriminating PD-L1 expression levels was relatively modest, with AUC values ranging from 0.637 to 0.705 based on ROC curve analysis. No statistically significant differences were observed in AUC values among the individual sequence models (all p-values > 0.05). The combined model achieved the highest AUC value (AUC = 0.809), and statistically significant differences were found between the combined model and each individual sequence model in evaluating PD-L1 expression in PCNSL. The AUC values, sensitivity, specificity, and Youden's index for the various models and the representative parameters in predicting high and low PD-L1 expression in PCNSL are reported in **Tables 4**-**5** and **Figure 4**. Representative MRI images of two PCNSL cases with high and low PD-L1 expression are shown in **Figure 5** and **Figure 6**, respectively.

**Table 4:** Logistic regression models in the prediction of PD-L1 expression in PCNSLs.

Model	Level	*P* value	AUC	Sen(%)	Spe(%)	P value^#^
T2WI model			0.637(0.548-0.719)	30.56	93.62	0.002
Intercept	-4.890	0.004				
Entropy	1.318	0.019*				
T1WI model			0.705(0.619-0.782)	75.00	61.70	0.011
Intercept	-5.111	0.024				
Interquartile Range	-0.522	0.045*				
Mean Absolute Deviation	1.669	0.041*				
Minimum	0.020	0.030*				
Variance	-0.029	0.052				
FLAIR model			0.665(0.577-0.745)	44.44	82.98	< 0.001
Intercept	-4.167	0.020				
P90	0.081	0.090				
Mean	0.482	0.063				
Median	0.124	0.078				
Root Mean Squared	-0.681	0.063				
T1WI+C model			0.683(0.596-0.762)	80.56	53.19	0.013
Intercept	0.916	0.232				
Range	-0.006	0.013*				
ADC model			0.652(0.564-0.734)	91.67	38.30	0.002
Intercept	0.459	0.422				
Range	-0.004	0.011*				
Combined model			0.809(0.731-0.873)	80.56	73.40	–
Intercept	-11.544	0.003				
T2WI_Entropy	1.280	0.062				
T1WI_Interquartile Range	-0.512	0.062				
T1WI_Mean Absolute Deviation	1.648	0.054				
T1WI_Variance	-0.029	0.064				
FLAIR_Median	0.022	0.002*				
T1WI+C _Maximum	-0.006	0.061				

AUC, the area under the curve; Sen, sensitivity; Spe, specificity; *Significant difference (*P* < 0.05); *P* value^#^ represents the *p* value of the DeLong test for comparing the AUCs of each individual sequence model with the combined model.

**Table 5:** Performance of parameter in the prediction of PD-L1 expression in PCNSLs.

Parameter	T2WI_Entropy	T1WI_Minimum	FLAIR_Median	T1WI+C_Range	ADC_Range
AUC (95% CI)	0.637 (0548-0.719)	0.682 (0.595-0.761)	0.650 (0.561-0.731)	0.683 (0.596-0.762)	0.652 (0.564-0.734)
Sen (%)	30.56	66.67	86.11	80.56	91.67
Spe (%)	93.62	67.02	50.00	53.19	38.30
YI	0.24	0.34	0.36	0.34	0.30
Cutoff	2.60	45.94	241.95	323.86	421.89

AUC, the area under the curve; Sen, sensitivity; Spe, specificity; YI, Youden's Index.

## Discussion

Immunotherapy targeting the PD-1/PD-L1 pathway has demonstrated considerable promise in lymphoma (13–16). Prior research has indicated that patients with elevated PD-L1 expression may derive greater benefit from this treatment strategy (4, 6). In the present study, our results show that the combined model of whole-tumor histograms features derived from multiparametric MRI demonstrates promising potential in predicting PD-L1 expression in PCNSL, which may assist in the non-invasive identification of patients who could benefit from immunotherapy.

This study aimed to investigate the correlation between basic clinicopathological features and PD-L1 expression in PCNSL. Our results indicate that age, gender, and Hans classification are not significantly associated with PD-L1 expression levels, in agreement with prior studies (6). Additionally, previous research has reported a significant correlation between the Ki-67 index, a nuclear antigen protein associated with cellular proliferation, and PD-L1 expression (17, 18). However, in contrast to these findings, our study reveals that there is no statistically significant difference in Ki-67 index between the high and low PD-L1 expression groups.

Our findings suggest that whole-tumor histogram data derived from conventional MRI sequences, including T2WI, T1WI, FLAIR, T1WI+C, and DWI, are statistically significant in relation to PD-L1 expression status in PCNSL. For instance, the Entropy values extracted from T2WI sequences were higher in PCNSL patients with high PD-L1 expression compared to those with low PD-L1 expression. This finding may reflect the increased tumor heterogeneity observed in tumors with higher PD-L1 expression. Previous studies have demonstrated that ADC values play a crucial role in evaluating tumor PD-L1 expression status, although the results have been inconsistent and controversial (19–24). Our preliminary results indicate that in the high PD-L1 expression group, both the maximum and range values derived from ADC sequence are lower. This observation is consistent with previous studies conducted in head and neck squamous cell carcinoma and cervical cancer (19–21). The binding of PD-1 to PD-L1 can induce T-cell apoptosis, enabling tumor cells to evade the immune surveillance, which subsequently promotes tumor proliferation and disease progression (25, 26). This mechanism may account for the significant correlation observed between high PD-L1 expression and lower ADC values.

Histogram analysis based on individual conventional MRI sequences shows limited diagnostic performance in assessing PD-L1 expression in PCNSL. Although the T1WI model exhibits a slightly higher AUC compared to other sequences, these differences are not statistically significant. Our findings indicate that a fusion model combining multiple conventional sequences offers superior diagnostic performance, which is in line with previous studies (9, 27). This improvement may be due to the complementary information provided by multiparametric MRI sequences, which enables a more comprehensive and holistic characterization of tumor heterogeneity. It is noteworthy that the sequences used in our study, including T2WI, T1WI, FLAIR, T1WI+C, and DWI, are standard MRI sequences acquired prior to tumor treatment. Therefore, the whole-tumor histogram model based on these conventional MRI sequences offers excellent clinical applicability. This approach, being non-invasive, economical, and efficient, may serve as a useful adjunct in the development of individualized immunotherapy strategies, pending further validation.

Our study has several limitations. First, being a retrospective analysis, it is susceptible to selection bias. Second, although the sample size in this study is relatively large considering the rarity of PCNSL, the data were obtained from a single center. Future multi-center studies with larger, independent cohorts are warranted to externally validate and further refine the model, which will enhance its robustness and clinical applicability. Third, manual slice-by-slice delineation of the tumor is relatively time-consuming; in future studies, automated segmentation methods based on deep learning should be explored to improve efficiency. Fourth, histogram parameters derived from VOI were not accurately correlated with histopathological slices from corresponding sites. Therefore, a further prospective imaging-pathology correlation method may be required to validate the relationship between MR parameters and PD-L1 expression. Finally, due to the lack of statistically significant differences in baseline clinical characteristics and conventional MRI features between PD-L1 expression subgroups in PCNSL, we were unable to construct a comprehensive visual nomogram incorporating additional variables.

## Conclusion

In this study, we explored the feasibility of developing a non-invasive imaging biomarker for predicting PD-L1 expression in PCNSL by extracting whole-tumor histogram features from conventional multiparametric MRI sequences. Compared to single-sequence models, the combined model exhibited superior diagnostic performance. This non-invasive approach holds potential as a supplementary tool to inform personalized immunotherapy decision-making in clinical practice.

## Data Availability

The original contributions presented in the study are included in the article/**Supplementary Material**. Further inquiries can be directed to the corresponding authors.
