# Supplementary material for: Evaluation of programmed cell death ligand-1 expression in primary central nervous system lymphoma using whole-tumor histogram analysis of multiparametric MRI: implications for immunotherapy selection
Source: Front Immunol. 2025 Dec 11;16:1676273. doi: 10.3389/fimmu.2025.1676273 (PMC12738945; doi:10.3389/fimmu.2025.1676273)
Supplement: Supplementary file 1 [file Table1.docx]

| Parameters | Low PD-L1 | High PD-L1 | *P* value | Parameters | Low PD-L1 | High PD-L1 | *P* value |
| --- | --- | --- | --- | --- | --- | --- | --- |
| **T2WI model** |  |  |  | Maximum | 158.50±41.87 | 146.08±21.20 | 0.250 |
| P10 | 93.58±31.97 | 104.20±30.76 | 0.154 | Mean Absolute Deviation | 10.15±3.42 | 8.82±2.01 | 0.033 |
| P90 | 205.15±46.36 | 218.50±51.57 | 0.246 | Mean | 99.78±16.65 | 102.22±13.30 | 0.259 |
| Energy (10^8^) | 4.21±5.79 | 3.65±6.41 | 0.354 | Median | 100.10±17.09 | 102.24±13.58 | 0.320 |
| Entropy | 2.89±0.40 | 3.07±0.34 | 0.026 | Minimum | 33.60±35.47 | 52.77±22.45 | 0.001 |
| Interquartile Range | 53.14±18.78 | 56.93±19.62 | 0.408 | Range | 124.90±58.16 | 93.31±33.13 | 0.002 |
| Kurtosis | 5.42±2.66 | 4.61±2.35 | 0.066 | Robust Mean Absolute Deviation | 6.68±2.17 | 5.92±1.44 | 0.059 |
| Maximum | 375.47±94.66 | 359.80±90.99 | 0.305 | Root Mean Squared | 100.80±16.67 | 102.91±13.27 | 0.308 |
| Mean Absolute Deviation | 35.42±12.08 | 36.33±10.44 | 0.617 | Skewness | -0.17±0.95 | -0.17±0.70 | 0.759 |
| Mean | 143.21±33.10 | 155.72±36.62 | 0.111 | Uniformity | 0.19±0.07 | 0.16±0.05 | 0.058 |
| Median | 135.58±33.30 | 149.11±35.81 | 0.086 | Variance | 203.60±162.20 | 140.92±65.18 | 0.018 |
| Minimum | -4.25±45.26 | 17.05±49.53 | 0.031 | **FLAIR model** |  |  |  |
| Range | 379.71±118.08 | 342.75±100.87 | 0.092 | P10 | 196.03±44.29 | 217.59±30.50 | 0.016 |
| Robust Mean Absolute Deviation | 22.91±8.20 | 24.17±7.95 | 0.489 | P90 | 299.53±39.33 | 316.74±36.50 | 0.045 |
| Root Mean Squared | 151.60±32.80 | 163.15±37.17 | 0.129 | Energy (10^8^) | 9.66±1.31 | 7.78±8.64 | 0.344 |
| Skewness | 0.83±0.68 | 0.71±0.74 | 0.300 | Entropy | 2.89±0.41 | 2.96±0.37 | 0.374 |
| Uniformity | 0.18±0.06 | 0.15±0.05 | 0.034 | Interquartile Range | 50.98±18.78 | 49.40±15.34 | 0.617 |
| Variance (10^3^) | 2.46±1.64 | 2.41±1.32 | 0.723 | Kurtosis | 6.37±4.20 | 5.50±3.28 | 0.328 |
| **T1WI model** |  |  |  | Maximum | 379.13±55.77 | 382.97±53.09 | 0.860 |
| P10 | 83.89±17.05 | 88.43±13.30 | 0.082 | Mean Absolute Deviation | 33.04±11.66 | 31.88±7.75 | 0.823 |
| P90 | 115.59±17.90 | 116.33±13.90 | 0.677 | Mean | 247.28±35.80 | 266.52±32.58 | 0.011 |
| Energy (10^8^) | 1.64±2.75 | 1.17±1.40 | 0.218 | Median | 249.03±36.25 | 268.33±34.30 | 0.008 |
| Entropy | 2.82±0.45 | 3.01±0.36 | 0.066 | Minimum | 19.20±82.06 | 48.05±85.17 | 0.078 |
| Interquartile Range | 15.70±4.98 | 13.88±3.45 | 0.040 | Range | 359.94±108.91 | 334.92±103.88 | 0.183 |
| Kurtosis | 5.89±5.09 | 4.63±2.90 | 0.046 | Robust Mean Absolute Deviation | 21.68±8.07 | 20.92±5.95 | 0.723 |
| Root Mean Squared | 251.75±35.03 | 270.07±32.55 | 0.013 | Uniformity | 0.10±0.05 | 0.09±0.02 | 0.127 |
| Skewness | -0.73±0.97 | -0.69±0.90 | 0.696 | Variance (10^3^) | 5.74±3.33 | 4.63±2.31 | 0.071 |
| Uniformity | 0.18±0.06 | 0.17±0.05 | 0.385 | **ADC model** |  |  |  |
| Variance (10^3^) | 2.19±1.63 | 1.91±0.93 | 0.843 | P10 | 51.79±21.32 | 54.10±20.04 | 0.522 |
| **T1+C model** |  |  |  | P90 | 174.37±46.63 | 166.26±41.07 | 0.402 |
| P10 | 103.71±23.15 | 101.36±21.22 | 0.811 | Energy (10^8^) | 2.25±2.67 | 1.78±2.64 | 0.109 |
| P90 | 294.32±73.83 | 277.57±46.45 | 0.063 | Entropy | 2.86±0.49 | 3.02±0.48 | 0.098 |
| Energy (10^8^) | 8.25±1.20 | 5.02±5.78 | 0.088 | Interquartile Range | 62.30±24.33 | 58.33±17.61 | 0.463 |
| Entropy | 3.57±0.31 | 3.67±0.16 | 0.105 | Kurtosis | 6.52±4.35 | 5.79±4.07 | 0.210 |
| Interquartile Range | 1116.88±47.23 | 104.49±33.11 | 0.111 | Maximum | 391.74±131.25 | 335.12±103.38 | 0.027 |
| Kurtosis | 2.52±2.36 | 2.28±0.48 | 0.751 | Mean Absolute Deviation | 39.91±14.32 | 36.19±9.47 | 0.183 |
| Maximum | 372.26±82.95 | 338.90±60.09 | 0.007 | Mean | 103.93±25.75 | 103.60±27.25 | 0.868 |
| Mean Absolute Deviation | 61.35±21.66 | 56.00±15.02 | 0.095 | Median | 91.72±26.41 | 94.51±28.02 | 0.482 |
| Mean | 205.42±48.11 | 194.77±30.06 | 0.114 | Minimum | -13.19±46.50 | 3.20±35.96 | 0.063 |
| Median | 212.85±54.49 | 201.71±36.13 | 0.170 | Range | 404.93±151.31 | 331.92±105.13 | 0.011 |
| Minimum | 24.63±32.00 | 35.77±26.05 | 0.071 | Robust Mean Absolute Deviation | 26.64±10.13 | 24.70±7.20 | 0.333 |
| Range | 347.63±93.17 | 303.14±65.97 | 0.002 | Root Mean Squared | 117.28±27.86 | 114.14±27.87 | 0.571 |
| Robust Mean Absolute Deviation | 47.62±18.60 | 42.94±12.80 | 0.114 | Skewness | 1.31±0.78 | 1.14±0.85 | 0.313 |
| Root Mean Squared | 218.09±51.96 | 205.98±32.35 | 0.075 | Uniformity | 0.19±0.07 | 0.16±0.06 | 0.100 |
| Skewness | -0.14±0.41 | -0.21±0.44 | 0.759 | Variance (10^3^) | 3.06±2.30 | 2.33±1.17 | 0.166 |

Table.S1 Comparisons of histogram features of multiparametric MRI in PCNSLs with low PD-L1 and high PD-L1 expression.
